# Contribution of Antibiotic Susceptibility Testing and CH Typing Compared to Next-Generation Sequencing for the Diagnosis of Recurrent Urinary Tract Infections Due to Genetically Identical Escherichia coli Isolates: a Prospective Cohort Study of Cystitis in Women

**DOI:** 10.1128/spectrum.02785-22

**Published:** 2023-07-11

**Authors:** Nicolas Vautrin, Kévin Alexandre, Martine Pestel-Caron, Estelle Bernard, Roland Fabre, Marie Leoz, Sandrine Dahyot, François Caron

**Affiliations:** a Univ Rouen Normandie, Univ Caen Normandie, INSERM, DYNAMICURE UMR 1311, Rouen, France; b Univ Rouen Normandie, Univ Caen Normandie, INSERM, DYNAMICURE UMR 1311, CHU Rouen, Department of infectious diseases, Rouen, France; c Univ Rouen Normandie, Univ Caen Normandie, INSERM, DYNAMICURE UMR 1311, CHU Rouen, Department of Bacteriology, Rouen, France; d CHU Rouen, Department of Bacteriology, Rouen, France; e Laboratoire d’Analyses Médicales, Elbeuf, France; Post Graduate Institute of Medical Education and Research

**Keywords:** antibiotic susceptibility profile, CH typing, cystitis, NGS, recurrence, typing, uropathogenic *Escherichia coli* (UPEC), urinary tract infection

## Abstract

Recurrent cystitis is a common disease in women, mainly due to uropathogenic Escherichia coli (UPEC). For decades, typing methods now considered obsolete suggested that relapse by the same clone is dominant over reinfection, most UPEC strains being otherwise fully susceptible to antibiotics. We aimed to update these data. Thanks to a prospective study over 17 months, we recruited 323 women with cystitis. Of these, 251 of them had sporadic infection and 72 had recurrence, with 2 to 9 episodes per patient for a total of 131 UPEC isolates and 145 UPEC pairs at patient level. Phylogroups B2 (52.4%) and D (14.1%) were overall dominant, as expected due to their particular urovirulence. CH typing identified 119 distinct profiles with no CH type particularly associated with recurrence. Relapse was attested by CH typing for only 30.6% (22 out of 72), with very diverse situations ranging from all episodes due to the same clone to alternating reinfections and relapses. Next-generation sequencing confirmed the clonality for all but two of the 145 UPEC pairs. Antibiotic resistance was common for recurrent cystitis isolates (only 25 [17.2%] out of 145 UPEC pairs were fully susceptible), allowing us to predict UPEC clonality. Indeed, antibiotic susceptibility profile matched CH typing for 104 (71.7%) pairs. Finally, we demonstrated a large genetic diversity among UPEC isolates responsible for cystitis in women, even in cases of recurrence for which reinfection appeared dominant over relapse. Recurrent cystitis appears to be a heterogeneous disease requiring tailored treatment and prevention.

**IMPORTANCE** More than half of women will experience cystitis during their lifetime. Among these women, 25% will experience a second episode within the following 6 months. It is epidemiologically important to discriminate relapses from reinfections. Relapse identification relies on long and laborious methods and might influence treatment. Therefore, the designation of time- and cost-effective strategies for this goal is of particular interest. Our work suggests using CH typing and antibiotic susceptibility profiles to type Escherichia coli, the main uropathogen.

## INTRODUCTION

Cystitis is by far the most common presentation of sex-linked urinary tract infections (UTI), affecting 40% of women during their lifetime (1), from teenage years to old age. Usually considered a benign but uncomfortable condition, cystitis induces a substantial health care burden, being one of the leading causes of antibiotic treatment in otherwise healthy women (2). Cystitis is a dichotomous disease: while three-quarters of affected women have only a few episodes (i.e., sporadic cystitis [SC]), the other quarter suffers from recurrent cystitis (RC), commonly defined as at least 2 episodes within 6 months or 3 episodes within 12 months (3). When highly frequent, RC strongly affects the quality of life as a real handicap, worsened by the risk of antibiotic resistance and treatment escape (4).

As noted in a 2021 review, “relatively little is understood about the infection cycle of RC in humans” (2), despite numerous *in vitro* studies focusing on bacterial virulence factors particularly for uropathogenic Escherichia coli (UPEC), the leading species that accounts for about 80% of total UTI cases. For decades, the ascending route of cystitis has been accepted, considering that uropathogens usually come from the reservoir of fecal microbiota and colonize the periurethral area before ascending into the bladder. However, recent data support a more multifaceted mechanism of RC depending on the patient, involving the fecal abundance of uropathogens, vaginal dysbiosis, and persistence of UPEC within bladder epithelial cells as quiescent intracellular reservoirs (QIR) (2). A better understanding of RC, in particular the real proportion of relapse by the same clone versus reinfection by another lineage, would be of great interest, conceptually and practically, with the goal to move more frequently toward tailor-made treatment.

Historical studies using pulsed-field gel electrophoresis (PFGE), now recognized as insufficiently discriminating (5), have reported that a large majority of RC cases are due to reinfection by the primary strain (6–8). At that time, comparing antibiotic susceptibility profiles (ASP) between UPEC strains was of poor utility in detecting clonal isolates, due to the low incidence of antibiotic resistance resulting in most lineages sharing an identical wild-type phenotype (9). Subsequently, molecular methods adapted to E. coli typing such as Clermont phylotyping and CH typing have been developed. Phylotyping is based on the presence or absence of four loci (*arpA*, *chuA*, *yjaA*, and TspE4.C2) and allows assignment of an E. coli strain to one of the 7 phylogroups (A, B1, B2, C, D, E, and F) (10). CH typing is based on sequence analysis of internal fragments of two highly polymorphic genes, *fumC* and *fimH* (11). These methods are now considered more reliable than PFGE to investigate the clonality of E. coli strains (12).

In this context, thanks to a large prospective regional cohort of patients with well-characterized UTI and to typing methods such as CH typing, we aimed to analyze the microbiological epidemiology of sporadic versus recurrent cystitis in women, with a particular attention to the diagnosis of UPEC relapse versus reinfection at patient level by different typing methods.

## RESULTS

### Cohort description.

During the 17-month period, 445 bacterial isolates were collected from 323 women with cystitis, of whom 251 were classified as having SC and 72 as having RC. Patients from the SC group were younger (mean age of 54.3 versus 58.6 years for RC group, *P* = 0.048) and had a lower prevalence (29.5% versus 65.3%, *P* < 0.001) of complicated cystitis (cystitis associated with urological abnormality, chronic renal failure, or immunodeficiency according to current French guidelines [13]) (see Table S1 in the supplemental material).

Overall, 265 isolates were collected among the 251 patients with SC (some patients had 2 episodes more than 6 months apart during the study) and 180 isolates were collected among the 72 patients with RC (2 to 9 episodes per patient). As shown in Table 1, more than three-quarters of isolates were identified as E. coli (*n *= 347) with a significantly higher representation in the SC group than the RC group (81.5% versus 72.8%, *P* = 0.039).

**TABLE 1:** Bacterial species identified for the 445 isolates collected from the 323 women with recurrent cystitis (RC) or sporadic cystitis (SC)^*a*^

Bacterial species	*n* (%) by group:		
	All isolates (*n* = 445)	SC isolates (*n* = 265)	RC isolates (*n* = 180)
**Escherichia coli**	**347 (78%)**	**216 (81.5%)**	**131 (72.8%)**
A	47 (13.5%)	26 (12%)	21 (16%)
B1	34 (9.8%)	18 (8.3%)	16 (12.2%)
B2	182 (52.4%)	119 (55.1%)	63 (48.1%)
C	16 (4.6%)	6 (2.8%)	10 (7.6%)
**D**	**49 (14.1%)**	**40 (18.5%)**	**9 (6.9%)**
E	3 (0.9%)	1 (0.5%)	2 (1.5%)
F	16 (4.6%)	6 (2.8%)	10 (7.6%)

**Klebsiella pneumoniae**	**19 (4.3%)**	**6 (2.3%)**	**13 (7.2%)**
Proteus mirabilis	14 (3.1%)	6 (2.3%)	8 (4.4%)
Citrobacter koseri	9 (2%)	7 (2.6%)	2 (1.1%)
Other *Enterobacterales* species	7 (1.6%)	5 (1.9%)	2 (1.1 %)

Non-*Enterobacterales* species	49 (11%)	25 (9.4%)	24 (13.3 %)

a Significant differences (*P* < 0.05) between groups are presented in bold.

### UPEC antibiotic susceptibility profile.

As shown in Table 2, there was among UPEC isolates an overall low rate of resistance (<5%) to fosfomycin, nitrofurantoin, pivmecillinam, amoxicillin-clavulanic acid, and third-generation cephalosporin (3GC). For these antibiotics, no significant differences in resistance rates were observed between SC and RC groups, except for 3GC (higher resistance rate in the RC group, mainly due to extended-spectrum beta-lactamase [ESBL] production). In contrast, resistance was common and significantly higher among RC than SC patients for amoxicillin, trimethoprim-sulfamethoxazole, and quinolones.

**TABLE 2:** Rates of resistance to different antibiotics among the 347 E. coli isolates collected from patients with sporadic cystitis (SC) or recurrent cystitis (RC)^*a*^

Antibiotic(s)	Resistance to antibiotic by group, no. positive/total no. (%)		
	All isolates (*n* = 347)	SC isolates (*n* = 216)	RC isolates (*n* = 131)
**Amoxicillin**	**170/347 (49%)**	**93/216 (43.1%)**	**77/131 (58.8%)**
Amoxicillin + clavulanic acid	14/345 (4.1%)	6/214 (2.8%)	8/131 (6.1%)
Pivmecillinam	10/334 (3%)	5/211 (2.4%)	5/123 (4%)
**Ceftriaxone**	**11/344 (3.2%)**	**3/216 (1.4%)**	**8/128 (6.2%)**
**Nalidixic acid**	**56/347 (16.1%)**	**21/216 (9.7%)**	**35/131 (26.7%)**
**Ciprofloxacin**	**40/347 (11.5%)**	**16/216 (7.4%)**	**24/131 (18.3%)**
**Trimethoprim + sulfamethoxazole**	**78/343 (22.7%)**	**30/214 (14%)**	**48/129 (37.2%)**
Nitrofurantoin	4/347 (1.2%)	2/347 (0.9%)	2/131 (1.5%)
Fosfomycin	3/342 (0.9%)	1/214 (0.5%)	2/128 (1.6%)

a Significant differences (*P* < 0.05) between groups are presented in bold.

### UPEC typing.

UPEC phylotyping revealed that more than half of the 347 isolates belonged to group B2 (182/347, 52.4%) followed by group D (49/347, 14.1%) (Table 1). There was no significant difference between SC and RC when phylogroups were considered separately, except for group D (18.5% versus 6.9%, respectively; *P* = 0.004). Phylogroups B2 and D, associated with uropathogenic strains (14), were more frequent in SC than RC (73.6% and 55.0%, respectively).

**FIG 1:**
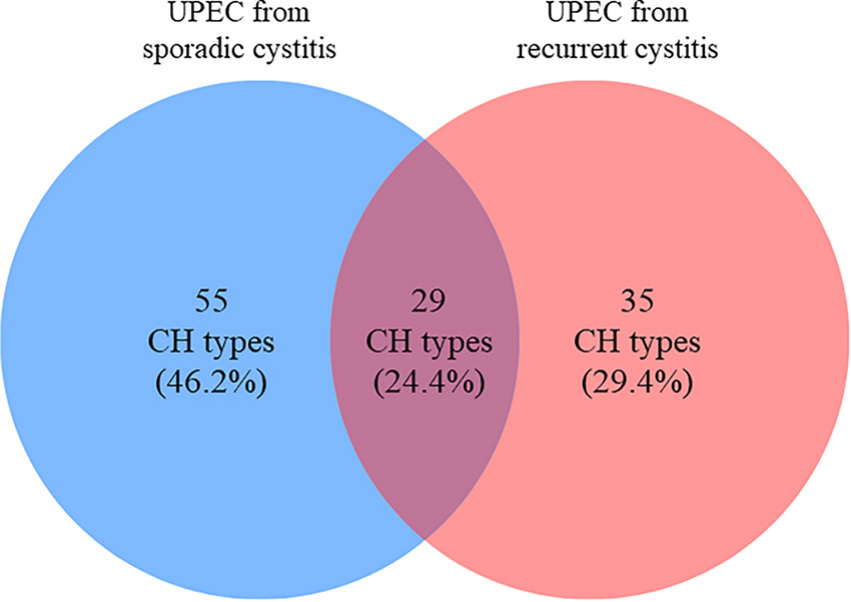
Venn diagram illustrating the repartition of the CH types for 347 UPEC isolates from sporadic or recurrent cystitis.

CH typing revealed a great genetic diversity, as 119 distinct CH types were identified for the 347 UPEC isolates. Five major CH types ([35_27], [14_27], [52_5], [11_54], and [40_30]) were shared by 104 isolates, accounting for 30.0% of the total set (Table S2). In contrast, 72 CH types (60.5%) were identified as singletons (i.e., in only one patient), in similar proportions in the SC group (43 singletons out of 84 CH types, 51.2%) and the RC group (29 out of 64, 45.3%). Ninety CH types were observed in only one of the two clinical groups, i.e., either in the SC group or in the RC group (Fig. 1). Simpson’s diversity indexes (DI) attested to this great diversity for the total cohort (DI = 0.976; 95% confidence interval [CI] = 0.970 to 0.982), as well as for the SC group (DI = 0.966; 95% CI = 0.955 to 0.977) and the RC group (DI = 0.979; 95% CI = 0.972 to 0.987).

**FIG 2:**
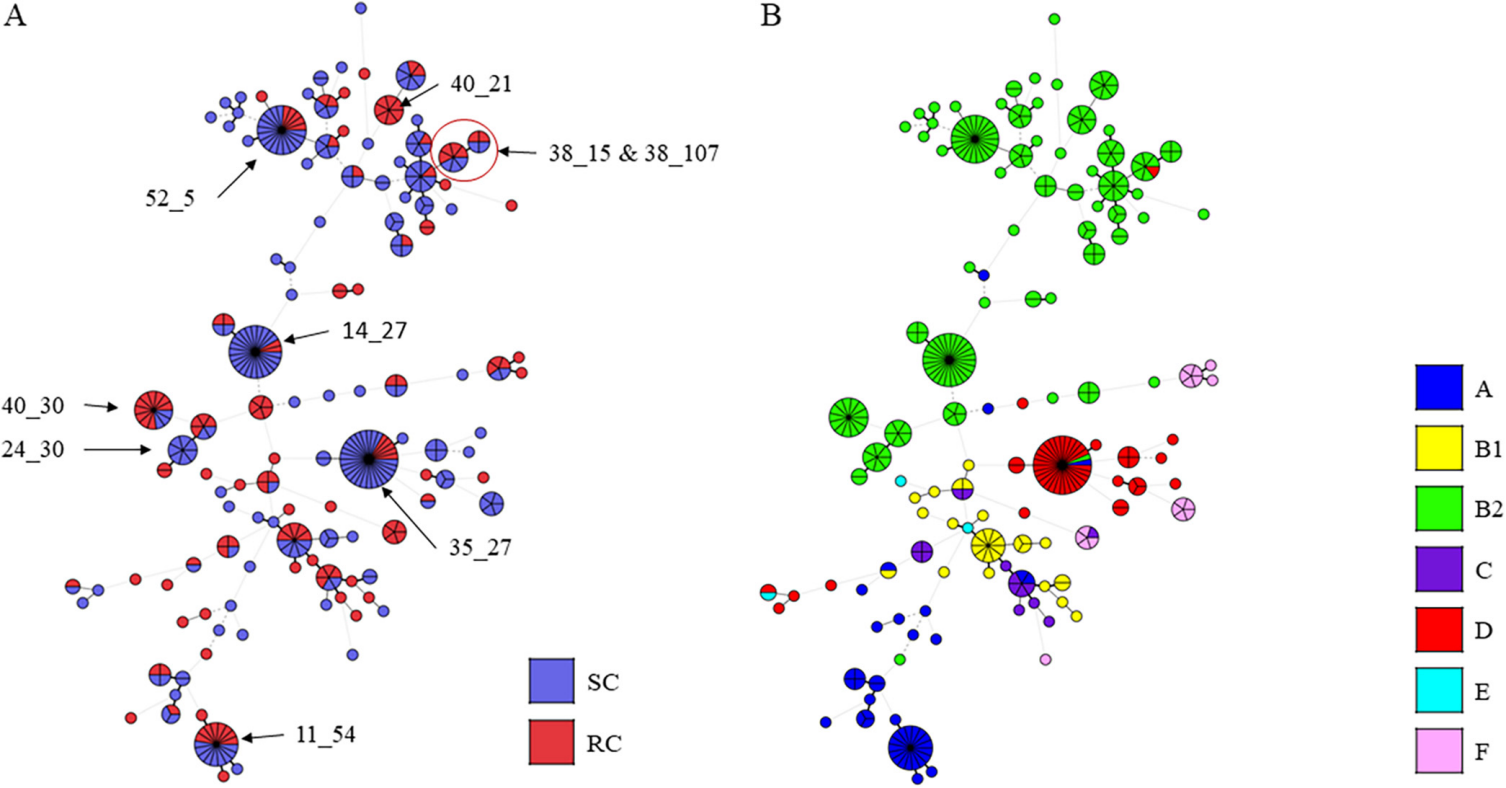
CH typing-based phylogeny of 347 E. coli isolates represented as minimum spanning trees according to clinical presentation (A) or phylogroup (B). Each circle represents a CH type, and each division represents an isolate. Length of strings represents the genetic distance between CH types. Major CH types were indicated on the tree as *fumC* allele number_*fimH* allele number. SC, sporadic cystitis; RC, recurrent cystitis.

The phylogenetic tree did not highlight any genetic links between CH types and clinical groups (Fig. 2A). In contrast, results of CH typing and phylotyping were overall consistent (Fig. 2B). Indeed, isolates belonging to the same phylogroup were assigned to genetically linked CH types. The only exception was for group F isolates, which did not cluster together on the tree.

### Longitudinal study of RC patients.

**FIG 3:**
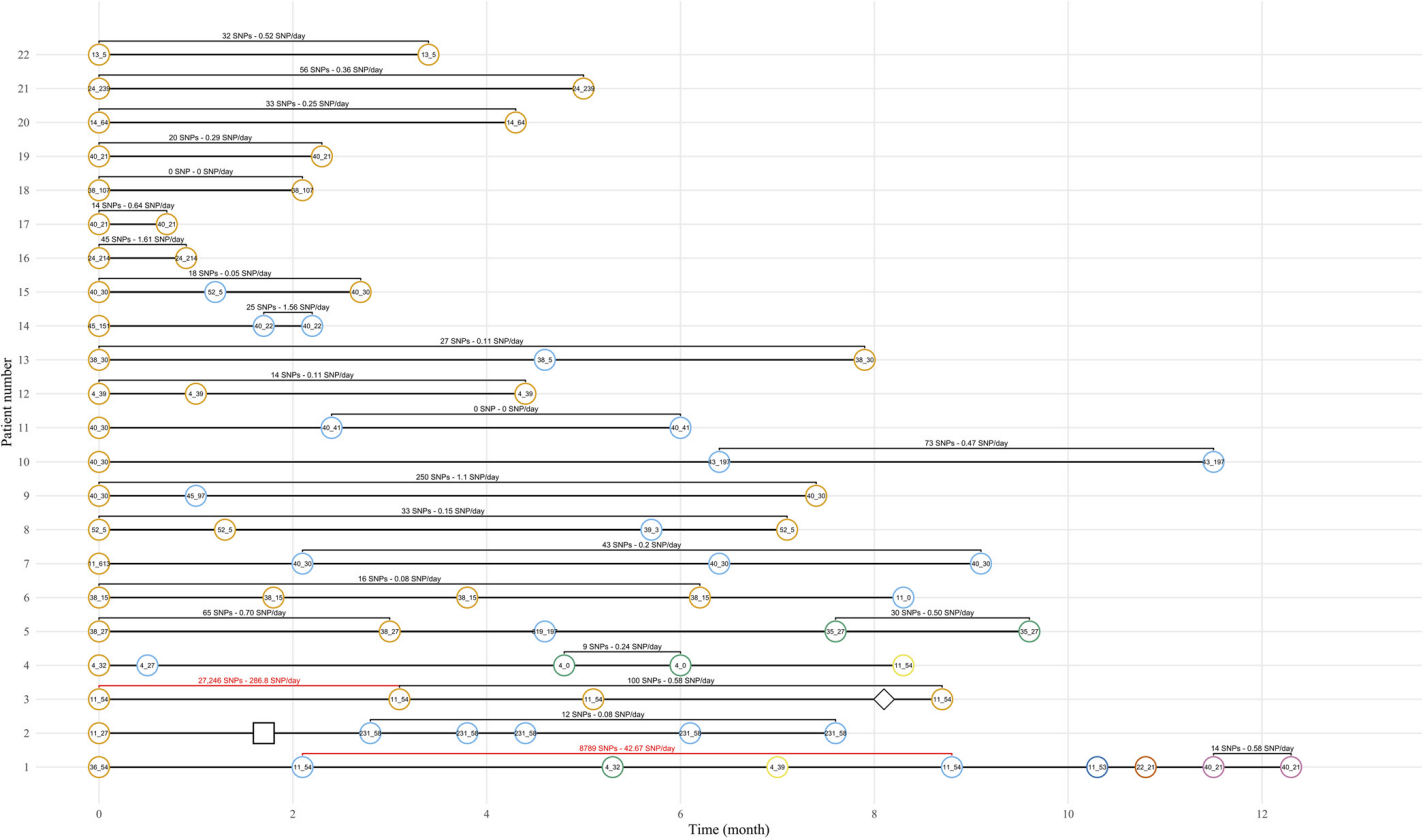
History of the 22 patients with relapse(s). Each line represents one patient, circles are for UPEC isolates, squares are for Enterococcus faecalis, and diamonds are for Klebsiella pneumoniae. Colors represents identical UPEC isolates for a given patient. Numbers in circles are CH types of the isolates.

As shown in Table 3, 72 patients suffered from RC during the study period, for a total of 180 episodes (separated by an average of 111 days). Forty patients (55.5%) presented at least two episodes with UPEC, of whom 22 patients (30.6%) had at least two episodes with UPEC assigned to the same phylogroup and CH type. Next-generation sequencing (NGS) analysis of the 35 suspected identical UPEC pairs (60 UPEC isolates) from these 22 patients revealed a small number of single nucleotide polymorphisms (SNPs) between isolates from most patients (0.39 SNPs/day on average, ranging from 0.00 to 2.15 SNPs/day). Of note, all UPEC isolates suspected to be identical by phylogroup and CH type were confirmed identical by SNP analysis (Table 3), except for two isolate pairs (46 SNPs/day and 286.8 SNPs/day). Figure 3 shows the UPEC history for each of the 22 patients, showing very diverse situations. Eight patients (patients 12 and 16 to 22) had only episodes with identical UPEC isolates, 11 patients (patients 2, 3, 6 to 11, and 13 to 15) were infected by 2 different UPEC isolates, 2 patients (patients 4 and 5) were infected by 3 different UPEC isolates, and 1 patient was infected by 5 different UPEC isolates.

**TABLE 3:** Descriptive table of recurrent cystitis group population^*a*^

Category	No. in recurrent cystitis group			
	Total patients	Total isolates	Total UPEC	Identical UPEC isolates
Overall	72	180	131	77
No case with UPEC	14	28	0	0
1 case only with UPEC	18	36	18	0
≥2 cases with UPEC				
All different by phylogroup/CH type	18	37	36	0
Some identical by phylogroup/CH type	22	79	77	60
Some identical by NGS	22	79	77	57

a UPEC, uropathogenic Escherichia coli; NGS, next-generation sequencing.

As shown in Table 4, ASP matched CH typing for 104 (71.7%) of the 145 UPEC pairs. Of note, while 51 (38.5%) out of the 131 UPEC isolates from the RC group displayed a wild-type phenotype, only 24 (16.5%) out of the 145 pairs shared this ASP.

**TABLE 4:** Intrapatient pairwise comparison between recurrent cystitis isolates in terms of antibiotic susceptibility profile (ASP) and CH typing

Result of ASP	Result of CH typing	
	Identical	Different
Identical pairs	33	25
(WT^*a*^ isolate pairs)	(15)	(10)
Different pairs	16	71

a WT, wild type.

## DISCUSSION

Distinguishing between reinfection and relapse helps physicians in the management of both uncomplicated and complicated RC (13). Indeed, RC may suggest an intrahost reservoir that could contribute to recurrence (15). Diagnosis of reinfection is easy when all subsequent episodes for a given patient are due to different bacterial species, but such events are uncommon: in our series of 72 patients suffering from RC, only 16 (22.2%) met this criterion. Indeed, UPEC accounted for 72.8% of the episodes of our RC patients, a percentage in phase with the literature data (16, 17). As reported by others (18, 19), UPEC isolates were less prevalent in RC than SC (72.8% versus 81.5%, *P* < 0.05), and this was correlated with a higher rate of complicated UTI among RC patients. This is consistent with the fact that urological abnormalities, which are the leading cause of complicated UTI, favor colonization and infection even by less urovirulent microbial species. As expected, we observed an underrepresentation of phylogroups B2 and D when pooled among RC patients compared to SC patients (55.0% versus 73.6%, *P* < 0.001). Indeed, these two groups are known to express more urinary virulence factors (14).

Thus, the major challenge in the diagnosis of reinfection versus relapse is to discriminate between UPEC recurrences. Due to increasing antibiotic resistance among *Enterobacterales* over recent decades, the wild-type ASP (i.e., no acquired resistance mechanism expressed) is now uncommon and thus ASP are more diverse. In our series, among the 145 pairs of UPEC isolates at a patient level, only 24 (16.5%) demonstrated a wild-type ASP. This dramatically illustrates the deleterious effects of iterative antibiotic treatments, particularly on the rate of resistance to 3GC, quinolones, and trimethoprim-sulfamethoxazole. Such a worrisome trend has made ASP an interesting tool for detecting clonality. In our work, ASP matched CH type for 71.7% of the UPEC pairs at a patient level. Among the discrepancies, 61.0% (25/41) were due to identical ASP corresponding in fact to different CH types, and 39.0% (16/41) were due to the same clone that diverged due to the expression of new antibiotic resistance over time under treatment pressure. Such evolution for urine as well as gut isolates has been recently longitudinally analyzed over 4 years in a patient suffering from RC due to a clonal UPEC lineage that presented rapid adaptation to iterative antibiotic exposure (20).

Five previous studies have analyzed clonality among UPEC isolates subsequently isolated from patients with RC using either pulsed-field gel electrophoresis or serotyping (6–8, 21, 22). All five have concluded a high rate of clonality (68% to 77%). Using more discriminant methods, we describe less than a third of patients having at least one pair of identical UPEC isolates among their different cystitis episodes: 30.6% according to CH typing and 29.2% by NGS. The CH typing was initially developed as an alternative method to multilocus sequence typing (11). To our knowledge, our study is the first to evaluate the relevance of CH typing to investigate UPEC isolates responsible for RC and discriminate between relapse and reinfection at the patient level. Its high performance suggests it could be a reasonable first-line alternative to more time-consuming and expensive NGS technologies to distinguish relapse from reinfection by UPEC.

NGS remains, however, the gold standard for confirming the clonality of isolates, although there are no internationally recognized standards for sequence and analysis quality and data interpretation (23). Interestingly, the delay between clinical episodes due to genetically identical UPEC isolates was long in our series (median time, 111 days) and thus strongly in favor of true relapse rather than antibiotic failure to eradicate the initial infection. Whether an E. coli isolate causing relapse persists within urothelium as a QIR and/or within digestive microbiota as a dominant *Enterobacterales* population needs to be prospectively studied at the patient level.

Our series shows an overall high diversity among UPEC isolates responsible for either RC or SC. These results are in line with a recent Danish study reporting an SNP-based phylogeny of 156 UPEC isolates which shows that RC isolates did not constitute distinct monophyletic clusters (24).

The strengths of our study were a recruitment of clinically well characterized cystitis patients and an exhaustive microbiological analysis. The dominant limitation was the relatively short period of time (17 months) of follow-up, whereas it would be interesting to follow bacterial evolution at patient level for a much longer time as performed by others (20).

Finally, we demonstrated a large genetic diversity among UPEC isolates from cystitis in women, even for those suffering from recurrent infection, while historical studies using less discriminatory typing methods had concluded a high rate of clonality for the latter. Thus, the physiopathology of recurrent cystitis seems more complex than previously assumed. This may have important consequences in terms of treatment and prevention. Due to the now high level of antibiotic resistance of UPEC responsible for RC, comparison of ASP of isolates represents a routine way to approach clonality at patient level. For a more precise analysis, CH typing could offer in our opinion at this time the best compromise between reliability, feasibility, and cost compared to NGS, in almost every laboratory.

## MATERIALS AND METHODS

### Ethics.

This study was part of an epidemiological study on community-acquired UTI founded by the French Ministry of Health and approved by the Medical Research Ethics Committee of the Rouen University Hospital (VITALE study, ClinicalTrials.gov identifier NCT02292160). Participating patients received an information letter and provided written informed consent.

### Patients.

Adolescent and adult women with community-acquired cystitis were prospectively included over a 17-month period (September 2015 to January 2017) by a dedicated staff in a laboratory serving outpatients that analyzes around 4,000 urine samples per year (Laboratoire d’Analyses de Biologie Médicale, Elbeuf, Normandy, France). Cases with negative urine culture or catheter-related infections were excluded. After the interview of the patient and, when necessary, of the family doctor (either by email or by phone), UTIs were classified as uncomplicated or complicated according to national guidelines (13). RC was defined as at least 2 episodes of cystitis within 6 months (3). Relapse was defined as a recurrence due to isolates identical by typing. Reinfection was defined as a recurrence due to genetically different isolates.

### Bacterial identification.

Identification to the species level was performed routinely using either a biochemical method (Vitek 2 system; bioMérieux, Marcy-l’Étoile, France) or matrix-assisted laser desorption ionization–time of flight mass spectrometry (Bruker, Billerica, MA, USA). After identification, isolates were stored at −80°C using a cryobead system (Technical Service Consultants Ltd., Heywood, United Kingdom). Duplicate specimens, defined as the same species isolated from a given patient within 2 weeks from the first positive culture, were excluded.

### Antimicrobial susceptibility testing of UPEC.

ASP were determined using the Vitek 2 automated system (AST-N372 card). Extended-spectrum beta-lactamase-producing isolates detected by Vitek 2 were confirmed by the double-disk synergy test following the recommendations of the European Committee on Antimicrobial Susceptibility Testing (EUCAST) (https://www.eucast.org).

### UPEC molecular typing.

DNA from UPEC isolates was extracted using the InstaGene Matrix kit (Bio-Rad, Hercules, CA, USA) according to the manufacturer’s recommendations after overnight culture at 37°C on ChromID CPS Elite (bioMérieux, Marcy-l’Étoile, France). All PCRs were performed using the GoTaq Green master mix (Promega, Madison, WI, USA).

Phylogroup assignment (A, B1, B2, C, D, E, and F) was performed using the revised Clermont method (10, 25–28) with minor modifications concerning primer amounts as recommended by the GoTaq manufacturer (see Tables S3 and S4 in the supplemental material).

CH (*fumC*_*fimH*) typing was performed as described by Weissman et al. (11) with minor modifications. PCR amplification was conducted under the conditions described by Tartof et al. (29, 30) for *fumC* (fumarate hydratase coding gene) and by Weissman et al. (11) for *fimH* (type 1 fimbrin d-mannose specific adhesion protein coding gene) amplification (Tables S3 and S4). PCR products were purified and sequenced using Sanger technology by Eurofins GATC (Constance, Germany). All PCRs for phylogroup assignment and CH typing were performed on a Veriti thermal cycler (Applied Biosystems, Foster City, CA, USA).

Allelic profiles and corresponding CH types were assigned using BioNumerics software (version 7.6; Applied System, Sint-Martens-Latem, Belgium) by using the databases of the online tool CHTyper 1.0 (accessed 29 January 2021) (31). All CH types were named in the format “*fumC* allele number_*fimH* allele number.” Phylogeny based on concatenated sequences of *fumC* and *fimH* was used to construct minimum spanning trees with BioNumerics software using the unweighted pair group method with arithmetic mean (UPGMA). Simpson’s diversity index (DI) (32) and associated confidence intervals (CIs) (33) were calculated using the online tool available at http://www.comparingpartitions.info/?link=Tool.

### NGS sequencing and bioinformatics.

UPEC isolates from a given RC patient assigned to the same phylogroup and the same CH type were suspected not to be genetically different and selected for further analysis using whole-genome NGS.

Overnight-shaking Trypticase soya cultures (Bio-Rad, Hercules, CA, USA) were centrifuged for 10 min at 2,000 × *g*. Bacterial pellets were resuspended in a mix of 100 μL of sterile phosphate-buffered saline (PBS), 100 μL of MagNA Pure bacterial lysis buffer (Roche Life Science, Penzburg, Germany), and 40 μL of a proteinase K solution at 20 mg/mL (Sigma-Aldrich, Saint Louis, MO, USA). Lysis mix was then heated to 65°C for 10 min and to 95°C for 10 min. Finally, bacterial lysates were frozen and then sent to the Mutualized Platform for Microbiology of the Pasteur Institute (P2M; https://research.pasteur.fr/en/team/mutualized-platform-for-microbiology/), which performed DNA purification on the MagNA Pure 96 system (Roche Diagnostics, Bâle, Switzerland), library preparation using the Nextera XT library kit (Illumina Inc., San Diego, CA, USA), and paired-end sequencing on the NextSeq 500 system (Illumina Inc.) with an average read length of 150 bp. The reads were demultiplexed using bcl2fastq v2.20.0 (emea.support.illumina.com/downloads/bcl2fastq-conversion-software-v2-20.html), quality controlled using fastq_info v2.0 (gitlab.pasteur.fr/GIPhy/fastq_info), and taxonomy controlled using Kraken v2.1.1 (github.com/DerrickWood/kraken2). Quality filtering was performed using fqCleanER v21.06 (gitlab.pasteur.fr/GIPhy/fqCleanER), and contaminant reads were removed using BBMap v38.94 SEAL (sourceforge.net/projects/bbmap/).

Intrapatient sequential UPEC isolates belonging to the same CH type and phylogroup were then pairwise compared with the first occurrence by whole-genome SNP analysis. Short reads from the initial occurrences were assembled by P2M using fq2dna v21.06 (gitlab.pasteur.fr/GIPhy/fq2dna), and contaminant reads were removed using BBMap v38.94 bbsplit (sourceforge.net/projects/bbmap/). These assemblies were then used as references for mapping the short reads from corresponding subsequent occurrences and SNP analysis using Snippy v4.3.6 (github.com/tseemann/snippy). A divergence rate (number of SNPs per day) was determined using the dates of isolate collection. Isolates with a divergence rate inferior to 3.5 SNPs/day were classified as identical according to the work of Petronella et al. (34) and considered responsible for a relapse.

### Data analyses.

All UPEC isolates from a given patient with recurrent cystitis were compared pair by pair. Pair numbers were determined using the following formula:

$$\frac{n!}{k!\left(n-k\right)!}$$

Statistical analyses were performed using R (v4.1.3; R Foundation, Vienna, Austria). Comparisons between proportions were performed using Pearson’s chi-square test or Fisher’s exact test for count data when possible. Means were compared using Wilcoxon rank sum test with continuity correction.

### Data availability.

The raw sequencing data and assembled genomes used in this study have been deposited in the Sequence Read Archive (SRA; https://www.ncbi.nlm.nih.gov/sra) under NCBI BioProject accession no. PRJNA900024. SRA and assembly accession numbers are listed in Table S5 in the supplemental material.
