# Supplementary material for: Contribution of Antibiotic Susceptibility Testing and CH Typing Compared to Next-Generation Sequencing for the Diagnosis of Recurrent Urinary Tract Infections Due to Genetically Identical Escherichia coli Isolates: a Prospective Cohort Study of Cystitis in Women
Source: Microbiol Spectr. 2023 Jul 11;11(4):e02785-22. doi: 10.1128/spectrum.02785-22 (PMC10433834; doi:10.1128/spectrum.02785-22)
Supplement: Supplemental file 1 — Tables S1 to S5. Download spectrum.02785-22-s0001.docx, DOCX file, 0.04 MB [file spectrum.02785-22-s0001.docx]

**Table S1: Demographics and patients characteristics.** Significant differences are presented in bold.

| Characteristics | Entire cohort (n=323) | Sporadic  Cystitis (n=251) | Recurrent Cystitis (n=72) |
| --- | --- | --- | --- |
| Age |  |  |  |
| **Mean (years)** | **55.3** | **54.3** | **58.6** |
| Range (years) | [15-95] | [15-93] | [16-95] |
| Between 15 and 65 years (percentage) | 64.7% | 67.3% | 55.5% |
| Risk factor for complicated UTI (percentage) |  |  |  |
| Any | 26.0% | 24.3% | 31.9% |
| Urinary tract abnormalities | 9.3% | 7.6 % | 15.3% |
| Chronic renal failure | 0.3% | 0% | 1.4% |
| Immunodeficiency | 2.8% | 2.8% | 2.8% |

**Table S2: CH-types of the 347 UPEC isolates from sporadic cystitis (SC) or recurrent cystitis (RC)**. CH-types are listed by decreasing frequency. CH types identified in both clinical groups (*i.e.* SC and RC) are presented in bold.

| CH type n (%) | All UPEC   (n = 347) | UPEC from SC  (n=216) | UPEC from RC  (n=131) |
| --- | --- | --- | --- |
| **35_27** | **29 (8%)** | **24 (11%)** | **5 (4%)** |
| **14_27** | **24 (7%)** | **22 (10%)** | **2 (2%)** |
| **52_5** | **21 (6%)** | **16 (7%)** | **5 (4%)** |
| **11_54** | **17 (5%)** | **9 (4%)** | **8 (6%)** |
| **40_30** | **13 (4%)** | **3 (1%)** | **10 (8%)** |
| **4_32** | **10 (3%)** | **5 (2%)** | **5 (4%)** |
| **24_9** | **8 (2%)** | **7 (3%)** | **1 (1%)** |
| **38_15** | **7 (2%)** | **3 (1%)** | **4 (3%)** |
| 40_21 | 7 (2%) | 0 (0%) | 7 (5%) |
| **24_10** | **6 (2%)** | **5 (2%)** | **1 (1%)** |
| 24_30 | 6 (2%) | 6 (3%) | 0 (0%) |
| **38_30** | **6 (2%)** | **2 (1%)** | **4 (3%)** |
| **4_39** | **6 (2%)** | **2 (1%)** | **4 (3%)** |
| **40_22** | **6 (2%)** | **4 (2%)** | **2 (2%)** |
| **13_5** | **5 (1%)** | **3 (1%)** | **2 (2%)** |
| **14_5** | **5 (1%)** | **4 (2%)** | **1 (1%)** |
| 231_58 | 5 (1%) | 0 (0%) | 5 (4%) |
| **4_0** | **5 (1%)** | **2 (1%)** | **3 (2%)** |
| **45_97** | **5 (1%)** | **2 (1%)** | **3 (2%)** |
| **11_0** | **4 (1%)** | **3 (1%)** | **1 (1%)** |
| **11_27** | **4 (1%)** | **2 (1%)** | **2 (2%)** |
| **14_64** | **4 (1%)** | **2 (1%)** | **2 (2%)** |
| **24_120** | **4 (1%)** | **3 (1%)** | **1 (1%)** |
| 32_41 | 4 (1%) | 4 (2%) | 0 (0%) |
| 35_47 | 4 (1%) | 4 (2%) | 0 (0%) |
| **38_107** | **4 (1%)** | **2 (1%)** | **2 (2%)** |
| **38_5** | **4 (1%)** | **3 (1%)** | **1 (1%)** |
| **4_27** | **4 (1%)** | **1 (0%)** | **3 (2%)** |
| **4_609** | **4 (1%)** | **1 (0%)** | **3 (2%)** |
| **40_41** | **4 (1%)** | **2 (1%)** | **2 (2%)** |
| 100_96 | 3 (1%) | 3 (1%) | 0 (0%) |
| **11_24** | **3 (1%)** | **2 (1%)** | **1 (1%)** |
| 24_1 | 3 (1%) | 3 (1%) | 0 (0%) |
| 24_103 | 3 (1%) | 3 (1%) | 0 (0%) |
| 65_32 | 3 (1%) | 3 (1%) | 0 (0%) |
| **7_54** | **3 (1%)** | **1 (0%)** | **2 (2%)** |
| 11_25 | 2 (1%) | 2 (1%) | 0 (0%) |
| 13_106 | 2 (1%) | 2 (1%) | 0 (0%) |
| 24_2 | 2 (1%) | 2 (1%) | 0 (0%) |
| 24_214 | 2 (1%) | 0 (0%) | 2 (2%) |
| 24_239 | 2 (1%) | 0 (0%) | 2 (2%) |
| 26_27 | 2 (1%) | 2 (1%) | 0 (0%) |
| **35_30** | **2 (1%)** | **1 (0%)** | **1 (1%)** |
| **36_48** | **2 (1%)** | **1 (0%)** | **1 (1%)** |
| 38_27 | 2 (1%) | 0 (0%) | 2 (2%) |
| 4_121 | 2 (1%) | 2 (1%) | 0 (0%) |
| 43_197 | 2 (1%) | 0 (0%) | 2 (2%) |
| 100_611 | 1 (<1%) | 0 (0%) | 1 (1%) |
| 103_9 | 1 (<1%) | 1 (<1%) | 0 (0%) |
| 108_0 | 1 (<1%) | 1 (<1%) | 0 (0%) |
| 11_? | 1 (<1%) | 1 (<1%) | 0 (0%) |
| 11_215 | 1 (<1%) | 1 (<1%) | 0 (0%) |
| 11_23 | 1 (<1%) | 1 (<1%) | 0 (0%) |
| 11_30 | 1 (<1%) | 1 (<1%) | 0 (0%) |
| 11_34 | 1 (<1%) | 0 (0%) | 1 (1%) |
| 11_41 | 1 (<1%) | 0 (0%) | 1 (1%) |
| 11_53 | 1 (<1%) | 0 (0%) | 1 (1%) |
| 11_566 | 1 (<1%) | 1 (<1%) | 0 (0%) |
| 11_608 | 1 (<1%) | 0 (0%) | 1 (1%) |
| 11_613 | 1 (<1%) | 0 (0%) | 1 (1%) |
| 11_86 | 1 (<1%) | 1 (<1%) | 0 (0%) |
| 1154_54 | 1 (<1%) | 0 (0%) | 1 (1%) |
| 13_201 | 1 (<1%) | 0 (0%) | 1 (1%) |
| 13_202 | 1 (<1%) | 1 (<1%) | 0 (0%) |
| 14_144 | 1 (<1%) | 0 (0%) | 1 (1%) |
| 14_2 | 1 (<1%) | 1 (<1%) | 0 (0%) |
| 14_54 | 1 (<1%) | 1 (<1%) | 0 (0%) |
| 182_168 | 1 (<1%) | 0 (0%) | 1 (1%) |
| 19_27 | 1 (<1%) | 0 (0%) | 1 (1%) |
| 22_21 | 1 (<1%) | 0 (0%) | 1 (1%) |
| 23_38 | 1 (<1%) | 0 (0%) | 1 (1%) |
| 23_86 | 1 (<1%) | 1 (<1%) | 0 (0%) |
| 24_102 | 1 (<1%) | 1 (<1%) | 0 (0%) |
| 24_118 | 1 (<1%) | 1 (<1%) | 0 (0%) |
| 24_24 | 1 (<1%) | 1 (<1%) | 0 (0%) |
| 24_608 | 1 (<1%) | 0 (0%) | 1 (1%) |
| 24_610 | 1 (<1%) | 1 (<1%) | 0 (0%) |
| 24_615 | 1 (<1%) | 1 (<1%) | 0 (0%) |
| 24_96 | 1 (<1%) | 1 (<1%) | 0 (0%) |
| 249_274 | 1 (<1%) | 1 (<1%) | 0 (0%) |
| 26_172 | 1 (<1%) | 1 (<1%) | 0 (0%) |
| 26_65 | 1 (<1%) | 0 (0%) | 1 (1%) |
| 319_197 | 1 (<1%) | 0 (0%) | 1 (1%) |
| 35_31 | 1 (<1%) | 1 (<1%) | 0 (0%) |
| 35_612 | 1 (<1%) | 1 (<1%) | 0 (0%) |
| 36_54 | 1 (<1%) | 0 (0%) | 1 (1%) |
| 36_617 | 1 (<1%) | 1 (<1%) | 0 (0%) |
| 36_93 | 1 (<1%) | 1 (<1%) | 0 (0%) |
| 37_27 | 1 (<1%) | 0 (0%) | 1 (1%) |
| 372_10 | 1 (<1%) | 1 (<1%) | 0 (0%) |
| 38_21 | 1 (<1%) | 1 (<1%) | 0 (0%) |
| 38_41 | 1 (<1%) | 1 (<1%) | 0 (0%) |
| 38_54 | 1 (<1%) | 1 (<1%) | 0 (0%) |
| 38_92 | 1 (<1%) | 1 (<1%) | 0 (0%) |
| 39_3 | 1 (<1%) | 0 (0%) | 1 (1%) |
| 4_24 | 1 (<1%) | 0 (0%) | 1 (1%) |
| 4_303 | 1 (<1%) | 0 (0%) | 1 (1%) |
| 4_31 | 1 (<1%) | 1 (<1%) | 0 (0%) |
| 4_34 | 1 (<1%) | 0 (0%) | 1 (1%) |
| 4_35 | 1 (<1%) | 0 (0%) | 1 (1%) |
| 4_366 | 1 (<1%) | 0 (0%) | 1 (1%) |
| 4_38 | 1 (<1%) | 0 (0%) | 1 (1%) |
| 4_43 | 1 (<1%) | 0 (0%) | 1 (1%) |
| 41_30 | 1 (<1%) | 1 (<1%) | 0 (0%) |
| 43_616 | 1 (<1%) | 1 (<1%) | 0 (0%) |
| 45_151 | 1 (<1%) | 0 (0%) | 1 (1%) |
| 45_189 | 1 (<1%) | 0 (0%) | 1 (1%) |
| 52_14 | 1 (<1%) | 1 (<1%) | 0 (0%) |
| 52_186 | 1 (<1%) | 1 (<1%) | 0 (0%) |
| 52_414 | 1 (<1%) | 1 (<1%) | 0 (0%) |
| 52_614 | 1 (<1%) | 1 (<1%) | 0 (0%) |
| 52_75 | 1 (<1%) | 1 (<1%) | 0 (0%) |
| 52_76 | 1 (<1%) | 1 (<1%) | 0 (0%) |
| 58_24 | 1 (<1%) | 0 (0%) | 1 (1%) |
| 6_23 | 1 (<1%) | 1 (<1%) | 0 (0%) |
| 6_31 | 1 (<1%) | 1 (<1%) | 0 (0%) |
| 67_222 | 1 (<1%) | 1 (<1%) | 0 (0%) |
| 8_31 | 1 (<1%) | 1 (<1%) | 0 (0%) |
| 95_32 | 1 (<1%) | 1 (<1%) | 0 (0%) |

**Table S3: List of primers used for phylotyping and CH typing.** bp = base pair

| PCR reaction | Primer ID | Target | Primer sequences | Amplicon size (bp) | Reference |
| --- | --- | --- | --- | --- | --- |
| Quadruplex | chuA.1b | *chuA* | 5′-ATGGTACCGGACGAACCAAC-3′ | 288 | Clermont *et al*. 2013 (11) |
|  | chuA.2b |  | 5′-TGCCGCCAGTACCAAAGACA-3′ |  | Clermont *et al.* 2000 (12) |
|  | yjaA.1b | *yjaA* | 5′-CAAACGTGAAGTGTCAGGAG-3′ | 211 | Clermont *et al*. 2013 (11) |
|  | yjaA.2b |  | 5′-AATGCGTTCCTCAACCTGTG-3′ |  | Clermont *et al*. 2013 (11) |
|  | TspE4C2.1b | TspE4.C2 | 5′-CACTATTCGTAAGGTCATCC-3′ | 152 | Clermont *et al*. 2013 (11) |
|  | TspE4C2.2b |  | 5′-AGTTTATCGCTGCGGGTCGC-3′ |  | Clermont *et al*. 2013 (11) |
|  | AceK.f | *arpA* | 5′-AACGCTATTCGCCAGCTTGC-3′ | 400 | Clermont *et al*. 2013 (11) |
|  | ArpA1.r |  | 5′-TCTCCCCATACCGTACGCTA-3′ |  | Clermont *et al.* 2004 (13) |
|  |  |  |  |  |  |
| Group E | ArpAgpeE.f | *arpA* | 5′-GATTCCATCTTGTCAAAATATGCC-3′ | 301 | Lescat *et al.* 2012 (15) |
|  | ArpAgpeE.r |  | 5′-GAAAAGAAAAAGAATTCCCAAGAG-3′ |  | Lescat *et al.* 2012 (15) |
|  |  |  |  |  |  |
| Group C | ArpAgpeC.f | *arpA* | 5′-AGTTTTATGCCCAGTGCGAG-3′ | 219 | Lescat *et al.* 2012 (15) |
|  | ArpAgpeC.r |  | 5′-TCTGCGCCGGTCACGCCC-3′ |  | Lescat *et al.* 2012 (15) |
|  |  |  |  |  |  |
| Internal control | trpBA.f | *trpA* | 5′-CGGCGATAAAGACATCTTCAC-3′ | 489 | Clermont *et al.* 2008 (14) |
|  | trpBA.r |  | 5′-GCAACGCGGCCTGGCGGAAG-3′ |  | Clermont *et al.* 2008 (14) |
|  |  |  |  |  |  |
| *fimH* typing | fimH.f | *fimH* | 5’-CACTCAGGGAACCATTCAGGCA-3’ | 975 | Weissman *et al.* 2012 (16) |
|  | fimH.r |  | 5’-CTTATTGATAAACAAAAGTCAC-3’ |  | Weissman *et al.* 2012 (16) |
|  |  |  |  |  |  |
| *fumC* typing | fumC.f | *fumC* | 5’-TCACAGGTCGCCAGCGCTTC-3 | 806 | Wirth *et al.* 2006 (18) |
|  | fumC.r |  | 5’-GTACGCAGCGAAAAAGATTC-3’ |  | Wirth *et al.* 2006 (18) |

**Table S4: PCRs mix compositions and reactions conditions**

| PCR Reaction | GoTaq® volume | Target gene | Primers amount | Extracted DNA volume | Final volume | PCR conditions |
| --- | --- | --- | --- | --- | --- | --- |
| Quadruplex* | 10µL | All | 50pmol | 2µL | 20µL | Denaturation 4min at 94°C, 30 cycles (5s at 94°C and 20s at 59°C), final extension step of 5min at 72°C |
| Group C* | 10µL | *arpA* | 20pmol | 2µL | 20µL | Same conditions t Quadruplex |
|  |  | *trpA* | 12.5pmol |  |  |  |
| Group E* | 10µL | *arpA* | 20pmol | 2µL | 20µL | Denaturation 4min at 94°C, 30 cycles (5s at 94°C and 20s at 57°C), final extension step of 5min at 72°C |
|  |  | *trpA* | 12.5pmol |  |  |  |
| *fimH* typing | 12,5µL | *fimH* | 25pmol | 5µL | 25µL | Denaturation 2min at 95°C, 30 cycles (30s at 94°C, 30s at 57°C and 1min at 72°C), final extension step of 5min at 72°C |
| *fumC* typing | 12,5µL | *fumC* | 12.5pmol | 5µL | 25µL | Denaturation 2min at 95°C, 30 cycles (1min at 95°C, 1min at 52°C, 2min at 72°C), final extension step of 5min at 72°C |

*PCR products were visualized by an electrophoresis performed in a 2% agarose gel with 0.005µg.mL^-1^ of ethidium bromide. Migrations were performed at 100V during 90min

**Table S5: Accession number of sequencing data (NCBI BioProject accession PRJNA900024).**

| **Biosample** | **Patient** | **Date** | **isolate** | **SRA accession** | **Genome accession*** |
| --- | --- | --- | --- | --- | --- |
| SAMN31676723 | PAT1 | 2015-12-07 | 2102 | SRR22252631 | JARUIQ000000000 |
| SAMN31676724 | PAT1 | 2016-06-30 | 2506 | SRR22252630 |  |
| SAMN31676725 | PAT1 | 2016-09-19 | 2630 | SRR22252619 | JAQQHM000000000 |
| SAMN31676726 | PAT1 | 2016-13-10 | 2681 | SRR22252608 |  |
| SAMN31676727 | PAT2 | 2016-02-25 | 2287 | SRR22252597 | JAQQHA000000000 |
| SAMN31676728 | PAT2 | 2016-03-25 | 2329 | SRR22252586 |  |
| SAMN31676729 | PAT2 | 2016-04-13 | 2378 | SRR22252577 |  |
| SAMN31676730 | PAT2 | 2016-06-04 | 2442 | SRR22252576 |  |
| SAMN31676731 | PAT2 | 2016-07-19 | 2526 | SRR22252575 |  |
| SAMN31676732 | PAT3 | 2015-09-15 | 2001 | SRR22252574 | JARUIR000000000 |
| SAMN31676733 | PAT3 | 2015-12-19 | 2133 | SRR22252629 | JAQQGV000000000 |
| SAMN31676734 | PAT3 | 2016-02-19 | 2272 | SRR22252628 |  |
| SAMN31676735 | PAT3 | 2016-06-07 | 2448 | SRR22252627 |  |
| SAMN31676736 | PAT4 | 2016-06-06 | 2444 | SRR22252626 | JAQQHG000000000 |
| SAMN31676737 | PAT4 | 2016-07-13 | 2517 | SRR22252625 |  |
| SAMN31676738 | PAT5 | 2015-12-28 | 2146 | SRR22252624 | JAQQGW000000000 |
| SAMN31676739 | PAT5 | 2016-03-30 | 2345 | SRR22252623 |  |
| SAMN31676740 | PAT5 | 2016-08-16 | 2568 | SRR22252622 | JAQQHK000000000 |
| SAMN31676741 | PAT5 | 2016-10-15 | 2711 | SRR22252621 |  |
| SAMN31676742 | PAT6 | 2016-02-18 | 2359 | SRR22252620 | JAQQHB000000000 |
| SAMN31676743 | PAT6 | 2016-04-12 | 2377 | SRR22252618 |  |
| SAMN31676744 | PAT6 | 2016-06-14 | 2466 | SRR22252617 |  |
| SAMN31676745 | PAT6 | 2016-08-25 | 2582 | SRR22252616 |  |
| SAMN31676746 | PAT7 | 2016-05-09 | 2419 | SRR22252615 | JAQQHD000000000 |
| SAMN31676747 | PAT7 | 2016-09-16 | 2627 | SRR22252614 |  |
| SAMN31676748 | PAT7 | 2016-12-08 | 2783 | SRR22252613 |  |
| SAMN31676749 | PAT8 | 2015-10-29 | 2400 | SRR22252612 | JAQQHC000000000 |
| SAMN31676750 | PAT8 | 2015-12-09 | 2405 | SRR22252611 |  |
| SAMN31676751 | PAT8 | 2016-06-01 | 2438 | SRR22252610 |  |
| SAMN31676752 | PAT9 | 2015-09-14 | 2003 | SRR22252609 | JAQQGS000000000 |
| SAMN31676753 | PAT9 | 2016-04-27 | 2409 | SRR22252607 |  |
| SAMN31676754 | PAT10 | 2016-02-06 | 2440 | SRR22252606 | JAQQHF000000000 |
| SAMN31676755 | PAT10 | 2016-11-05 | 2749 | SRR22252605 |  |
| SAMN31676756 | PAT11 | 2016-07-08 | 2513 | SRR22252604 | JAQQHI000000000 |
| SAMN31676757 | PAT11 | 2016-10-25 | 2713 | SRR22252603 |  |
| SAMN31676758 | PAT12 | 2015-09-15 | 2002 | SRR22252602 | JAQQGR000000000 |
| SAMN31676759 | PAT12 | 2015-10-15 | 2030 | SRR22252601 |  |
| SAMN31676760 | PAT12 | 2016-01-28 | 2211 | SRR22252600 |  |
| SAMN31676761 | PAT13 | 2016-05-04 | 2536 | SRR22252599 | JAQQHJ000000000 |
| SAMN31676762 | PAT13 | 2016-12-30 | 2782 | SRR22252598 |  |
| SAMN31676763 | PAT14 | 2016-18-08 | 2571 | SRR22252596 | JAQQHL000000000 |
| SAMN31676764 | PAT14 | 2016-09-03 | 2595 | SRR22252595 |  |
| SAMN31676765 | PAT15 | 2016-11-03 | 2739 | SRR22252594 | JAQQHN000000000 |
| SAMN31676766 | PAT15 | 2017-01-24 | 2840 | SRR22252593 |  |
| SAMN31676767 | PAT16 | 2016-06-22 | 2484 | SRR22252592 | JAQQHH000000000 |
| SAMN31676768 | PAT16 | 2016-07-20 | 2528 | SRR22252591 |  |
| SAMN31676769 | PAT17 | 2016-05-09 | 2432 | SRR22252590 | JAQQHE000000000 |
| SAMN31676770 | PAT17 | 2016-05-31 | 2435 | SRR22252589 |  |
| SAMN31676771 | PAT18 | 2016-02-12 | 2257 | SRR22252588 | JAQQGY000000000 |
| SAMN31676772 | PAT18 | 2016-04-16 | 2384 | SRR22252587 |  |
| SAMN31676773 | PAT19 | 2015-10-31 | 2070 | SRR22252585 | JAQQGT000000000 |
| SAMN31676774 | PAT19 | 2016-01-08 | 2165 | SRR22252584 |  |
| SAMN31676775 | PAT20 | 2015-12-12 | 2110 | SRR22252583 | JAQQGU000000000 |
| SAMN31676776 | PAT20 | 2016-04-22 | 2399 | SRR22252582 |  |
| SAMN31676777 | PAT21 | 2016-01-04 | 2155 | SRR22252581 | JAQQGX000000000 |
| SAMN31676778 | PAT21 | 2016-06-06 | 2443 | SRR22252580 |  |
| SAMN31676779 | PAT22 | 2016-02-17 | 2270 | SRR22252579 | JAQQGZ000000000 |
| SAMN31676780 | PAT22 | 2016-04-18 | 2392 | SRR22252578 |  |

* Genome assembly was performed for the 25 initial occurrences of relapsing UPEC strains.
